# Improving fatigue behavior of CAD/CAM-ground 4YSZ via finishing, polishing, and surface treatments

**DOI:** 10.1590/0103-644020256592

**Published:** 2025-12-08

**Authors:** Kétlin Fagundes Teixeira, Luiza Freitas Brum Souza, Pablo Machado Soares, Renatta Wrasse Temp, Cornelis Johannes Kleverlaan, João Paulo Mendes Tribst, Gabriel Kalil Rocha Pereira, Luiz Felipe Valandro, Ana Carolina Cadore-Rodrigues

**Affiliations:** 1 Graduate Program in Oral Science, Faculty of Dentistry, Federal University of Santa Maria (UFSM), Santa Maria, Rio Grande do Sul State, Brazil; 2 Department of Dental Materials Science, Academic Centre for Dentistry Amsterdam (ACTA), Universiteit van Amsterdam and Vrije Universiteit, Amsterdam, Noord-Holland, The Netherlands

**Keywords:** Air-abrasion, Flexural strength, Low-fusing glaze, Surface characteristics, Yttrium stabilized zirconia

## Abstract

It evaluated the effect of an internal finishing and polishing protocol, combined with surface treatments, on the fatigue flexural strength and topography of 4YSZ ceramic after CAD/CAM grinding. Ceramic bars (18×5.2×2.4 mm; IPS e.max ZirCAD MT, Ivoclar) were assigned in six groups (n=15) based on two factors: 'Surface condition' - with (POL) or without (non-POL) internal finishing and polishing using F, FF diamond burs and polishing tips (Optragloss, Ivoclar); and 'Surface treatments' - CTRL (no treatment), AB (air-abrasion with 45 µm aluminum oxide), or GLZ (glaze spray). Surface roughness was measured, and a cyclic flexural three-point bending fatigue test (20 Hz, 10,000 cycles/step, step size 25 MPa starting at 75 MPa) was conducted. Fractographic and topographic analyses were also performed. Fatigue data were analyzed by Two-Way ANOVA, Kaplan-Meier, Mantel-Cox (Log-Rank), and Weibull analyses, while roughness data underwent Two-Way ANOVA and Tukey's tests. 'Surface condition' significantly influenced fatigue strength (F=18.38, p<0.05), while 'Surface treatment' and the interaction between factors were not significant (p>0.05). POL groups exhibited higher fatigue behavior than non-POL groups: CTRL (POL vs. non-POL, p = 0.000), AB (POL vs. non-POL, p = 0.049), GLZ (POL vs. non-POL, p = 0.004). Roughness showed POL consistently smoother than non-POL in every comparison: non-POL-CTRL > POL-CTRL, non-POL-AB > POL-AB, and non-POL-GLZ > POL-GLZ. Topographic analysis indicated that air abrasion produced uniform surfaces, while glaze partially filled in defects. Fractures originated from surface defects in tensile regions. Internal finishing and polishing after CAD/CAM grinding enhance the flexural strength of 4YSZ, with air-abrasion and glaze yielding comparable fatigue performance.

## Resumo

Este estudo avaliou o impacto de um protocolo de acabamento interno e polimento após o fresamento CAD/CAM, combinado com tratamentos de superfície, sobre a resistência à fadiga flexural e a topografia da cerâmica 4YSZ. As barras cerâmicas (18 × 5,2 × 2,4 mm; IPS e.max ZirCAD MT, Ivoclar AG) foram divididas em seis grupos (n= 15) com base em dois fatores: 'Condição da superfície' - com (POL) ou sem (non-POL) acabamento interno e polimento utilizando fresas diamantadas F, FF e pontas de polimento (Optragloss, Ivoclar AG); e 'Tratamentos de superfície' - CTRL (sem tratamento), AB (jateamento com óxido de alumínio de 45 µm) ou GLZ (spray de glaze). A rugosidade da superfície foi medida e um teste de fadiga cíclica de flexão em três pontos (20 Hz, 10.000 ciclos/passos, tamanho de passo 25 MPa iniciando a 75 MPa) foi realizado. Também foram feitas análises fractográficas e topográficas. Os dados de fadiga foram analisados por ANOVA de dois fatores e testes de Kaplan-Meier, enquanto os dados de rugosidade foram analisados por ANOVA de dois fatores e testes de Tukey. A 'Condição da superfície' influenciou significativamente a resistência à fadiga (F= 18,38, p < 0,05), enquanto 'Tratamento de superfície' e a interação entre os fatores não foram significativos (p > 0,05). Os grupos POL apresentaram comportamento de fadiga superior ao grupo non-POL: CTRL (POL vs. non-POL p= 0,000), AB (POL vs. non-POL p= 0,049), GLZ (POL vs. non-POL p= 0,004). A rugosidade foi consistentemente mais suave no grupo POL do que no non-POL em todas as comparações: non-POL-CTRL > POL-CTRL, non-POL-AB > POL-AB, e non-POL-GLZ > POL-GLZ. A análise topográfica indicou que o jateamento com ar produziu superfícies uniformes, enquanto o glaze preencheu parcialmente os defeitos. As fraturas originaram-se de defeitos superficiais nas regiões de tração. O acabamento interno e o polimento após o fresamento CAD/CAM melhoram a resistência flexural da 4YSZ, sendo que o jateamento com ar e o glaze proporcionaram desempenho de fadiga comparável.

**Figure:**
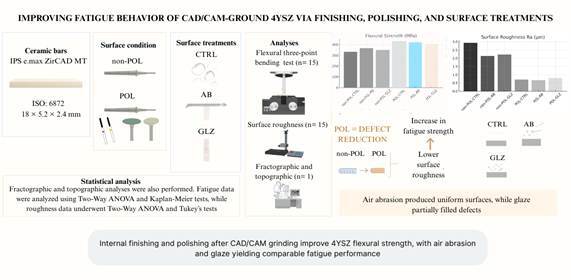

## Introduction

Yttrium-stabilized zirconia (YSZ) ceramics are commonly employed in prosthetic restorations for full arch, posterior crowns, fixed dental prostheses, and infrastructures^1^ ^,^ ^2^. Monolithic zirconia restorations offer aesthetic properties, biocompatibility, and notable mechanical performance^1^ ^,^ ^2^ ^,^ ^3^, showcasing a 98% survival rate after five years in monolithic crowns^4^. The manufacturing of zirconia restorations was made feasible through advancements in computer-aided design/computer-aided manufacturing (CAD/CAM) technology^5^ ^,^ ^6^. This process involves processing dental model data through CAD software to design a restoration precisely. The design is then transferred to the CAM module, where computerized milling sculpts the material in a subtractive process to achieve the final form^6^.

The milling process promotes an increase in surface roughness as well as surface and subsurface damage in the ceramic restoration^3^ ^,^ ^7^. Failures in this material arise from milling tools, resulting in restorations characterized by a topography marked by populations of defects^3^ ^,^ ^7^, especially on the bonding surface^8^. This issue is critical due to the concentration of tensile stresses, which result in failures that reduce the longevity of the restoration^8^. Furthermore, CAD/CAM milling results in a weakening of zirconia's strength, leading to a 40% decrease in material flexural strength^5^.

Protocols aimed at minimizing or removing internal superficial defects may improve the performance of ceramic materials. Studies have shown that a three-step polishing system (finishing tip, polishing tip, and nylon brush with diamond paste) following internal adjustments of zirconia restorations significantly reduces surface roughness, resulting in a smoother, more reliable surface^9^ ^,^ ^10^. Another previous study observed that removing 80 µm of the surface after CAD/CAM milling is necessary to eliminate both surface and subsurface defects^5^. This removal was achieved using silicon carbide paper^5^, which is impractical for internal crown surfaces in clinical settings. Additionally, the procedure was done before sintering, which could lead to internal and marginal misfit of the crowns.

Furthermore, polishing tips after internal adjustments of zirconia restorations have been shown to reduce surface roughness, thereby promoting a smoother surface^9^ ^,^ ^10^. To this end, establishing a comprehensive protocol for finishing and polishing zirconia could be beneficial in reducing defect populations, especially when starting from a situation with a higher concentration of defects. From this perspective, a more detailed and systematic approach is needed, such as a protocol utilizing fine and extra-fine diamond burs in combination with polishing tips. Aiming to address a gap in the literature regarding defect reduction strategies, the present study proposes an original and unprecedented protocol. This strategy could be effective in minimizing milling defects and enhancing the mechanical performance of zirconia restorations.

Regarding surface treatments to improve the adhesion of YSZ restorations, air abrasion with alumina particles (coated or not with silica) to create micromechanical retention remains the most widely used method^11^. However, especially in the case of 4YSZ, which exhibits a phase transformation mechanism only marginally^1^ ^,^ ^12^ ^,^ ^13^ ^,^ ^14^ ^,^ ^15^, air abrasion can increase the number of defects and a decrease in strength^14^ ^,^ ^16^. To overcome this limitation, studies have suggested chemically activating the surface by coating it with a low-fusion vitreous ceramic, using the glaze spray technique^16^ ^,^ ^17^ ^,^ ^18^. This is considered an effective alternative, offering a surface rich in silica and stable adhesion^19^. This material also exhibits a healing effect, filling microfissures and porosities, which results in a smoother surface and crack-resistant characteristics^16^ ^,^ ^17^. Glaze spray can cover defects caused by milling burs, improving stress distribution and fatigue resistance of 4YSZ^17^. However, defects originating from milling burs persist on the surface even after the application of glaze spray^17^. Thus, the combination of a protocol to reduce CAD/CAM surface defects, along with surface treatments, can promote a more favorable condition for enhanced 4YSZ mechanical behavior.

Considering the predictability and longevity of restorations, fatigue mechanical tests aim to predict material behavior through cyclic loading. These tests seek to replicate the behavior of materials or restorations under conditions similar to those found in a clinical environment, specifically under intermittent cyclic loading^8^, where materials are subjected to both chemical and mechanical stresses. Therefore, the present study aims to evaluate the effect of an internal finishing and polishing protocol, after in-laboratory CAD/CAM grinding and surface treatments, on the fatigue flexural strength and topographical characteristics of 4YSZ. The hypotheses assumed were that the finishing and polishing protocol, as well as the application of glaze spray as a surface treatment, would improve the fatigue flexural strength of 4YSZ. The hypotheses of this study were: 1) the finishing and polishing protocol (POL) would improve the flexural fatigue strength of 4YSZ, and 2) the application of glaze spray as a surface treatment would enhance the flexural fatigue strength of 4YSZ.

## Materials and methods

The general description (manufacturers, batch number, and composition) of the materials used in the present study is presented in Table 1.

**Table 1:** List of materials used in the study: commercial name, manufacturer, batch number, and composition based on the manufacturer's information.

Commercial name	Manufacturer (Batch number)	Composition
IPS e.max ZirCAD MT	Ivoclar AG (X16331)	ZrO_2_ (86.0 - 93.5 wg%);
		Y_2_O_3_(>6.5% - ≤ 8.0 wg%);
		HfO_2_ (≤ 5.0 wg%);
		Al_2_O_3_ (≤ 1.0 wg%);
		and other oxides (≤1.0 wg%)
VITA Akzent Plus	VITA Zahnfabrik (E92800)	Amorphous glassy substance (silica-based material)
Aluminum oxide	Ivoclar AG (68680)	Aluminum oxide particles (45 μm)
Diamond bur #F	KG Sorensen (036137)	Diamond grit size 46 μm and stainless steel
Diamond bur #FF	KG Sorensen (2202)	Diamond grit size 30 μm and stainless steel
Optragloss System	Ivoclar AG (ZL09P6)	Dark-blue and Light-blue tips: synthetic rubber, diamond granulate, titanium dioxide, and stainless steel;
12S cylinder pointed bur	Sirona Dental (M02225/M01241)	-

### Study design

**Figure 1:**
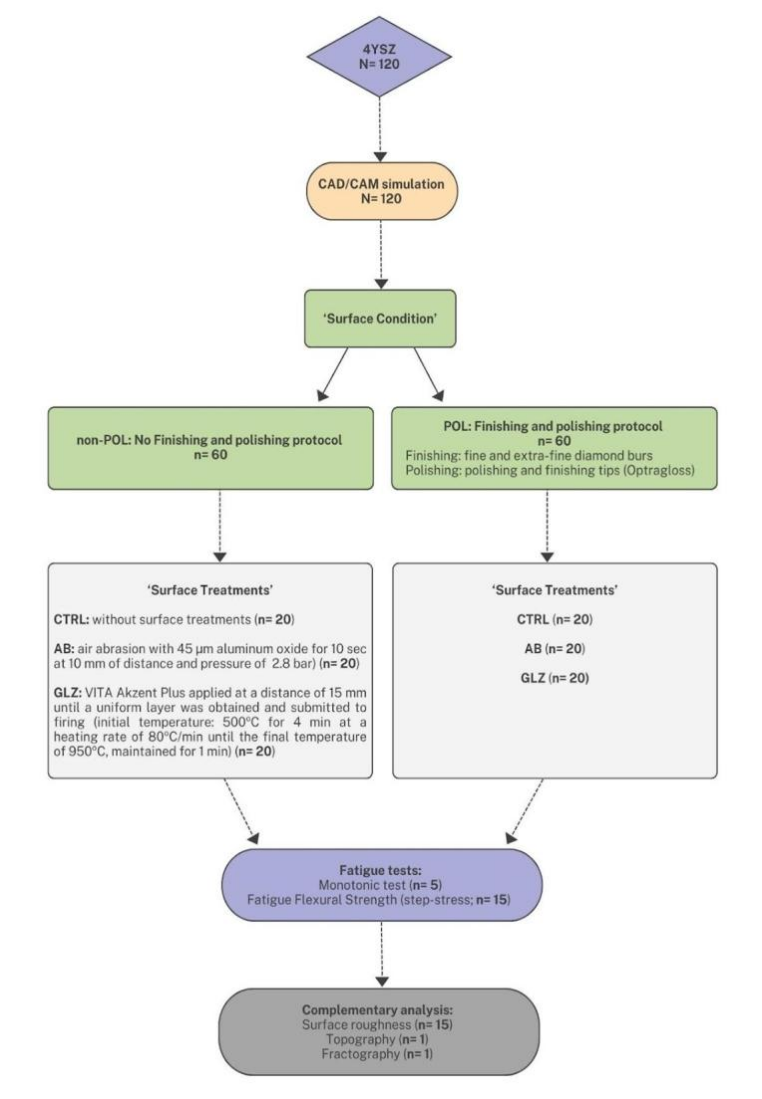
Flowchart illustrating the study design.

This in vitro study was designed with six experimental groups, considering two factors: surface condition and surface treatments. The surface condition was defined as either POL, using a finishing procedure with F and FF diamond burs followed by polishing with polishing tips, or non-POL (unfinished). The surface treatments included CTRL (no treatment), AB (air abrasion with 45µm aluminum oxide), and GLZ (glaze spray application), as presented in Table 2 and Figure 1.

The primary response variables assessed were fatigue flexural strength (FS) in MPa and the number of cycles to failure in fatigue (CFF). The sample size of 15 specimens per group was determined based on assumptions established in prior studies, ensuring adequate statistical power for Weibull modulus analysis^12^. This approach was employed to guarantee that the number of specimens would be sufficient to yield reliable results and accurately interpret the variability in fatigue resistance among different treatments.

Additionally, surface roughness measurements (Ra and Rz parameters in µm), surface topography, and fractographic features of failed specimens were examined using scanning electron microscopy (SEM).

**Table 2:** Study design.

Groups	Surface condition	Surface treatments
non-POL-CTRL	CAD/CAM grinding	Control
non-POL-AB		Air abrasion
non-POL-GLZ		Glaze spray application
POL-CTRL	CAD/CAM grinding + Finishing and polishing protocol*	Control
POL-AB		Air abrasion
POL-GLZ		Glaze spray application

*Finishing: fine and extra-fine diamond burs / Polishing: polishing and finishing tips of the Optragloss kit

### Specimen preparation - Bar-shaped specimen production

4YSZ ceramic blanks (18 × 98.5 mm; IPS e.max ZirCAD MT, Ivoclar AG, Schaan, Liechtenstein) were used to prepare bar-shaped specimens according to ISO 6872-2015. For this, the blanks were sectioned using a handpiece and a diamond disc into approximately 20 mm × 20 mm blocks. Subsequently, they were ground using a polishing machine with silicon carbide papers of #400 grit under refrigeration (EcoMet/AutoMet 250, Buehler, Lake Bluff, Illinois, United States) and sectioned using a precision cutting machine with a diamond blade (ISOMET 1000, Buehler, Lake Bluff, United States) to manufacture 90 bar-shaped specimens. Following this, the specimens were polished with silicon carbide paper of 600 and 1200 grit to achieve their pre-sintering dimensions of 21 × 5.4 × 2.8 mm, as measured with a digital caliper (Absolute 500-196-20, Mitutoyo, Kawasaki, Kanagawa, Japan).

### Specimen preparation - CAD/CAM grinding simulation

All bars were submitted to the CAD/CAM grinding, which was performed using CAD/CAM system burs (12S cylinder pointed bur, CEREC inLab, Sirona Dental, Charlotte, North Carolina, United States), following the procedure outlined by Cadore-Rodrigues et al.^17^. For the grinding, the CAD/CAM system bur was adapted to an electric motor handpiece operating at up to 30,000 rpm (W&H Dentalwerk, Bürmoos, Salzburg, Austria). To achieve this, the specimens were marked with graphite and placed on a glass plate using double-sided tape. Grinding was then conducted using circular movements performed by a trained operator, applying gentle manual pressure with oscillatory movements until the complete removal of the graphite ensured that the entire surface was ground. The burs were replaced for each group.

Previous studies suggest that the zirconia roughness values after CAD/CAM milling are 1.8 μm for Ra^20^ ^,^ ^21^. Thus, after the CAD/CAM grinding, all specimens were measured using a contact profilometer (SJ-410, Mitutoyo, Kawasaki, Kanagawa, Japan) to obtain samples with Ra = 2.2 before sintering and Ra = 1.8 after sintering. Specimens that did not meet these averages during pre-sintering were discarded, and new ones were fabricated. Although a trained operator performed the procedure and roughness values were controlled, our study observed roughness parameters with an average Ra of 2.4 post-sintering, using the CAD/CAM system bur. Following the CAD/CAM grinding, the bars' side edges underwent a brief grinding process for 5 seconds using #1200 grit SiC paper to produce a chamfer ranging from 0.09 mm to 0.15 mm in width (ISO 6872-2015). The dimensions of each chamfer were meticulously examined using a stereomicroscope at 50× magnification (Discovery V20, Carl Zeiss, Göttingen, Lower Saxony, Germany). Any identified disparities resulted in the replacement of the affected bar.

### Specimen preparation - Sintering

The specimens were sintered in a specific furnace (Vita Zyrcomat 6000 MS, Vita Zahnfabrik, Bad Säckingen, Baden-Württemberg, Germany) following the manufacturer's recommendations. The standard sintering cycle involves heating at a rate of 10 ºC/min until reaching 900 ºC, with a hold time of 30 minutes at that temperature. The temperature then increases at a rate of 3.3 ºC/min until it reaches 1500 ºC, where it is maintained for 120 minutes. Afterward, the cooling process occurs until the temperature reaches 900 and 300 ºC, resulting in a final post-sintering dimension of 18 × 5.2 × 2.4 mm.

### Specimen preparation - Finishing and polishing protocol

After sintering, half of the specimens were assigned to the finishing and polishing protocol (Table 2, Figure 1). Primarily, the surfaces with the CAD/CAM grinding were marked with graphite for standardization purposes. Then, the finishing procedure was performed using a fine diamond bur (#3216F - KG Sorensen, Cotia, São Paulo, Brazil), followed by an extra-fine diamond bur (#3216 FF - KG Sorensen) with oscillatory movements until complete removal of the graphite. Each bur was dedicated to every five specimens to mitigate wear-related influences^22^. Following this, the polishing protocol was performed according to Zucuni et al.^21^ using polishing tips from a polishing kit (Optragloss, Ivoclar AG, Barueri, São Paulo, Brazil). The dark-blue and light-blue tips of the polishing system were used for 25 s each, applying a standardized force of 3.9 N, as measured by a weighing scale^23^. The thickness was measured using a digital caliper (Absolute 500-196-20, Mitutoyo) before and after the application of the finishing and polishing protocol. The difference between the average measurements indicated a reduction of 80 µm.

### Surface Treatments

Before the surface treatments, all specimens were cleaned in an ultrasonic bath (model 1440 D, Odontobras, Ribeirão Preto, São Paulo, Brazil) containing distilled water for 5 minutes. Then, they were submitted to the surface treatments according to Table 2 and Figure 1 (control - no treatment, air abrasion with 45 µm aluminum oxide, or glaze spray application). The thickness of the glaze layer was measured using a digital caliper (Absolute 500-196-20, Mitutoyo). A comparison before and after the glaze spray application revealed an average increase of 30 µm in thickness.

### Roughness analysis

A micrometric surface roughness analysis was performed using a contact profilometer (SJ-410, Mitutoyo), by ISO 21920:2021 standards. The measurements were performed after the surface treatments of all specimens had been completed. Six measurements (three along the x-axis and three along the y-axis) were performed using a cut-off (λϲ) of 0.8 mm and a sampling length of 2 mm, considering the Ra (average surface roughness) and Rz (maximum peak-to-valley height) parameters.

### Monotonic test

To determine the fatigue parameters, a previous monotonic test (n = 5) was performed on a universal testing machine (DL-1000 Emic, EMIC, São Paulo, Brazil) at a crosshead speed of 1 mm/min, until failure, at the center of the bar. The specimens were tested using a three-point bending setup, with the treated surface positioned facing down on a specific jig with supported rollers (Ø = 2.0 mm) set 12 mm apart, and loading was applied by a third roller (Ø = 2.0 mm). An adhesive tape (110 μm) was placed on the upper surface of the specimen to prevent fragment scattering and surface damage upon contact, as well as between the specimen and the support rollers to reduce contact stress concentration. Consequently, the tape was replaced for each specimen^24^. The resulting data (in Newtons) was used to calculate the flexural strength (in MPa) using the following formula:

$$FS=\;\frac{3PL}{{2bh}^{2}}$$

in which P is the load for failure in Newtons, L is the test span (mm), b is the specimen width (mm), and h is the specimen thickness (mm) (ISO 6872-2015).

### Fatigue flexural strength (three-point bending test)

The cyclic fatigue test (n= 15) was conducted using a mechanical testing machine (Instron Corporation, Norwood, Massachusetts, United States) with the same three-point bending setup and methods previously described (item 2.5). The mean monotonic flexural strength of all groups (non-POL-CTRL: 514 MPa; non-POL-AB: 467 MPa; non-POL-GLZ: 470 MPa; POL-CTRL: 471 MPa; POL-AB: 501 MPa; POL-GLZ: 456 MPa) was used as a reference to determine the fatigue parameters for the fatigue test. The test was initiated with an initial stress level of 75 MPa (~15% of the monotonic test), a stress increment of 25 MPa (~5% of the monotonic test) applied every 10,000 cycles, and a frequency of 20 Hz^25^. All specimens were tested until the fracture occurred. For statistical analysis, the data for flexural fatigue strength (in MPa) and the number of cycles to failure (CFF) were recorded.

### Fractographic analysis

After the fatigue test, all specimens were examined under a stereomicroscope (Discovery V20, Carl Zeiss), and representative specimens (n=1) were chosen from each group for fractographic analysis. The specimens underwent cleaning in an ultrasonic bath (1440 D, Odontobras) with distilled water for 5 min and were gold-sputtered for Scanning Electron Microscopy analysis (SEM - VEGA3, Tescan, Brno, South Moravian, Czech Republic). Magnifications of 500× and 1000× were employed to identify the crack origin, specifically confirming that the crack originated at the tensile surface of the specimens. The SEM observations were performed with the following operational parameters: magnification of 500× and 1000×, voltage of 20 kV, working distance (WD) of 15 mm, and spot size of 46 nm.

### Topographic analysis

To access topographic characteristics in response to the finishing and polishing protocol and different surface treatments, additional specimens for each group (n=1) were prepared, cleaned in an ultrasonic bath (1440 D, Odontobras) with distilled water for 5 min, and coated with a gold-palladium alloy. Subsequently, the ceramic surfaces were analyzed at 700× magnification using SEM (Sigma 300 VP, Carl Zeiss, Oberkochen, Baden-Württemberg, Germany). The SEM observations were performed with the following operational parameters: magnification of 700×, voltage of 1 kV, WD ranging from 6.6 to 6.9 mm, and aperture size of 30 µm.

### Data analysis

Fatigue data of FS and CFF were submitted to a Two-Way analysis of Variance (Two-Way ANOVA). Additionally, a survival analysis for fatigue data was performed using Kaplan-Meier and Mantel-Cox (Log-Rank) tests (α= 0.05; SPSS Version 21, IBM Analytics, Chicago, Illinois, United States). Furthermore, FS and CFF data were subjected to Weibull statistical analysis to describe the Weibull modulus, using SuperSMITH Weibull 4.0k-32 software (Wes Fulton, San Francisco, California, United States). This analysis aimed to characterize the distribution of fatigue data, providing comprehensive insight into the mechanical reliability of the material in question.

As the roughness data assumed a parametric and homoscedastic distribution indicated by Shapiro-Wilk and Levene tests (p > 0.05), Two-way ANOVA (Analysis of Variance) and Tukey’s post-hoc tests (α= 0.05) were conducted using SPSS version 21 (IBM Analytics). Fractography and topography were qualitatively and descriptively analyzed to assess the topographic pattern of the surface of the study groups and analyze the fractographic characteristics, aiming to observe the induced effects on the ceramic material.

## Results

A power estimation was performed (G*Power software 3.1.9.6, Fraz Faul, Kiel, Germany) using a one-way ANOVA post hoc power analysis based on α = 0.05, sample size = 90, and effect size = 0.47, derived from the FS means and the mean standard deviation. The power was calculated with a noncentrality parameter λ= 20.22, critical F= 2.32, numerator df= 5, denominator df= 84, estimated in 1-β err prob= 0.94 (94%), indicating that the study had an adequate chance of identifying statistically significant differences between the groups in fatigue tests.

According to two-way ANOVA, the surface roughness was significantly influenced by the factors 'surface condition' and 'surface treatments', both individually (p < 0.05, F= 490.64; p < 0.05, F= 10.43, respectively) and in combination (p < 0.05, F= 11.44). The finishing and polishing protocol reduced the roughness values when comparing each surface treatment in POL and non-POL conditions (non-POL-CTRL > POL-CTRL; non-POL-AB > POL-AB; non-POL-GLZ > POL-GLZ). Besides, the surface treatments (AB and GLZ) promoted similar roughness values, regardless of the previous surface condition (POL or non-POL). However, in non-POL conditions, both surface treatments (AB and GLZ) resulted in inferior roughness compared to the CTRL group, while both treatments were similar to CTRL when considering the POL condition (Table 3).

**Table 3:** Results of fatigue tests by means of Kaplan-Meier and Mantel-Cox (Log-Rank) tests (mean and respective 95% confidence intervals for flexural strength - FS (MPa) and number of cycles for failure - CFF) and Weibull modulus with respective 95% confidence intervals for fatigue data (FS and CFF). Results of surface roughness parameters (Ra and Rz in µm - mean and standard deviation) by means of Two-way ANOVA and Tukey’s post-hoc tests.

Groups	Fatigue data		Weibull Modulus		Surface roughness	
	FS (MPa)	CFF	FS	CFF	Ra	Rz
non-POL-CTRL	320 (290 - 349)^D^	102,752 (91,205 - 114,300) ^D^	6.68 (4.27 - 9.72)^A^	5.60 (3.56 - 8.13)^A^	2.94 (0.33) ^A^	15.83 (23.38)^A^
non-POL-AB	365 (322 - 407)^BC^	119,504 (102,887 - 136,120)^BC^	5.90 (3.68 - 8.81)^A^	4.73 (2.94 - 7.09)^A^	2.14 (0.24) ^B^	12.18 (23.37)^B^
non-POL-GLZ	348 (320 - 375)^CD^	113,855 (102,418 - 125,292)^CD^	8.74 (5.5 - 12.78)^A^	6.95 (4.34 - 10.29)^A^	2.22 (0.73) ^B^	12.51 (22.81)^B^
POL-CTRL	431 (387 - 475)^A^	144,260 (128,596 - 159,925)^A^	5.95 (3.83 - 8.54)^A^	5.66 (3.63 - 8.15)^A^	0.71 (0.07)^C^	3.96 (0.42)^C^
POL-AB	421 (360 - 483)^A^	140,984 (119,835 - 162,133)^A^	4.32 (2.76 - 6.35)^A^	4.09 (2.59 - 5.97)^A^	0.67 (0.10)^C^	3.72 (0.48)^C^
POL-GLZ	405 (366 - 443)^AB^	133,621 (119,278 - 147,964)^AB^	6.79 (4.30-9.95)^A^	6.43 (3.99 - 9.60)^A^	0.80 (0.11)^C^	5.15 (0.76)^C^

**Figure 2:**
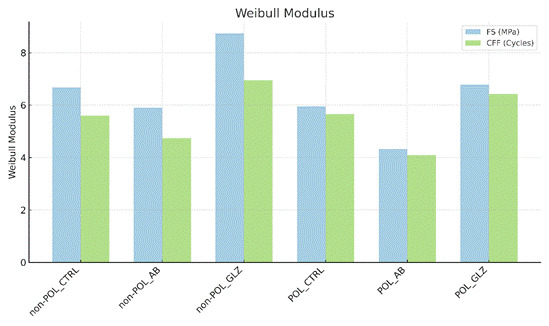
Weibull survival analysis plot showing the failure distribution and reliability of the 4YSZ.

Regarding the fatigue data from the two-way ANOVA, FS and CFF were significantly influenced by the factor 'surface condition' (p < 0.05, F= 18.38), but not by the factor 'surface treatments' (p > 0.05, F= 0.43) and in combination (p > 0.05, F= 1.09). According to Kaplan-Meir and Mantel-Cox, the POL-AB group had the highest FS and CFF values, followed by the POL-GLZ group and POL-CTRL, which showed similar values between them. Meanwhile, the non-POL-AB, non-POL-GLZ, and non-POL-CTRL groups had lower flexural strength values compared to their counterparts that received the POL condition (Table 3). The Weibull modulus showed statistically similar values across the groups (Table 3 and Figure 2), indicating consistent structural reliability among all the analyzed groups. This finding suggests that, despite differences in fatigue resistance, the materials exhibited similar levels of reliability in their structural performance. In other words, although the fatigue resistance varied between groups, the overall reliability of the materials, as assessed by the Weibull modulus, remained comparable across the different treatments.

**Figure 3:**
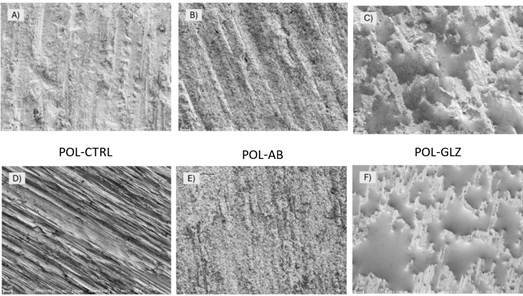
Topographic analysis at 700× magnification was performed on representative SEM images of ceramic surfaces after each condition. The CAD/CAM grinding induced surface alterations (non-POL-CTRL group), notably exhibiting grooves attributed to the grinding bur. The finishing and polishing protocol (POL-CTRL group) did not eliminate these defects but resulted in less aggressive ones. Regarding the surface treatments, air abrasion particles eroded the defects (likely decreasing the high peaks), leading to a smoother and more uniform surface (non-POL-AB and POL-AB), with the POL-AB group showing reduced grooves due to the POL condition. Conversely, GLZ covered some surface defects but was unable to uniformly fill the entire surface, leaving exposed areas (non-POL-GLZ and POL-GLZ), which could be more easily smoothed after the POL condition.

**Figure 4:**
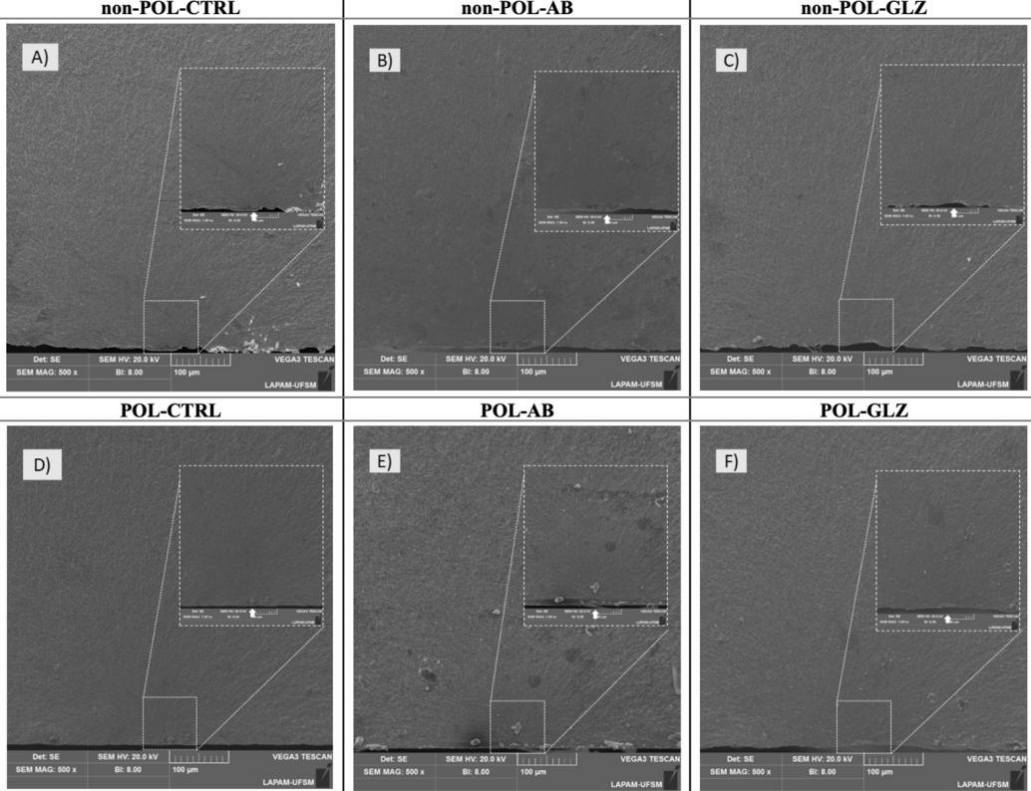
SEM fractographic images represent the zone of tensile stress concentration at 500x and 1000x magnification. The onset of failure, originating from surface defects due to the area subjected to fatigue testing, is indicated by the solid white arrow.

Comparing the topography of the non-POL-CTRL and POL-CTRL groups in Figure 3A and Figure 3D, it is evident that surface defects become less severe and pronounced after the finishing and polishing protocol. However, grooves are still visible on the surface even after the surface treatments with AB (Figure 3B and 4F) and GLZ (Figure 3C and 4F). Regarding surface treatments, each method produced distinct topographies due to its specific mechanism of action. Air abrasion induces surface erosion, leading to a more uniform and standardized distribution of defects in both POL and (Figure 3E) non-POL conditions (Figure 3B), whereas the glaze covers certain surface defects, leaving some areas uncovered in both POL (Figure 3C) and non-POL conditions (Figure 3F). Significantly, the topography of 4YSZ was less aggressive, with more uniformly distributed defects after surface treatments in the POL condition compared to the non-POL condition, attributed to the preceding finishing and polishing protocol.

**Figure 5:**
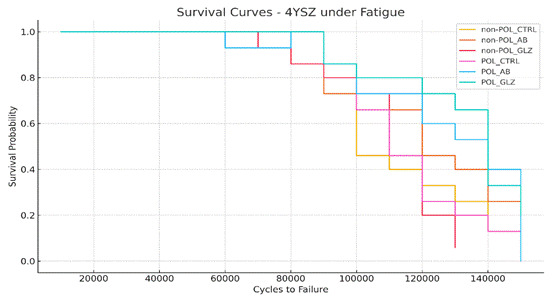
Kaplan-Meier survival plot illustrating the survival probability of the sample over time.

Fractographic analysis revealed that all fractures originated from surface defects in the specimens' regions subjected to tensile stress concentration (Figure 4A, 5B, 5C, 5D, 5E, 5F).

## Discussion

Since all groups in the POL condition demonstrated increased fatigue performance, the first hypothesis that the finishing and polishing protocol would improve flexural fatigue strength was accepted. Conversely, the second hypothesis that glaze application as a surface treatment would enhance the flexural fatigue strength of 4YSZ was rejected because there was no difference between the surface treatments in both the non-POL and POL conditions.

Surface defects generated during CAD/CAM milling, especially in areas subjected to tensile stress, play a critical role in the mechanical performance of zirconia. In high-translucency 4YSZ^17^, which contains a larger fraction of the cubic phase, crack propagation is more likely to occur because this phase does not undergo transformation toughening due to the absence of a stress-induced martensitic transformation layer^12^ ^,^ ^14^ ^,^ ^15^. Consequently, there is no energy dissipation mechanism to counteract crack growth, making internal flaws more critical^26^. CAD/CAM milling introduces surface and subsurface flaws, such as microcracks and irregularities, which act as stress concentrators and weaken the material, leading to premature failure^7^ ^,^ ^8^ ^,^ ^17^ ^,^ ^27^. The implementation of a controlled internal polishing protocol may help mitigate such defects by reducing local stress concentrations and promoting a more uniform stress distribution, which could delay crack initiation and ultimately improve the material's overall mechanical performance.

Corroborating with these findings, the results of our study demonstrated that a finishing and polishing protocol significantly reduced surface roughness (Ra and Rz parameters) and improved the material's flexural strength (Table 3). Additionally, the survival analysis (Table 4 and Figure 5) showed that the POL groups had a higher survival rate compared to the non-POL groups. At 375 FS/130,000 CFF (Table 4), the POL-CTRL group exhibited a 73% survival rate, while only about 13% of specimens survived in the non-POL-CTRL group. This increased strength and survival rate can be explained by removing 80 µm of the surface with the finishing and polishing protocol, which smoothed out the defects caused by the CAD/CAM grinding, resulting in a uniform surface pattern (Figure 3D). It corroborates with a prior study that removed a similar thickness of the surface, eliminating both surface and subsurface defects, resulting in improved mechanical performance of zirconia^21^. The two-step protocol, using fine and extra-fine diamond burs along with polishing tips, proved effective in mitigating the impact of these milling-induced defects, ultimately enhancing the material’s strength.

**Table:** 

Groups	Flexural strength / Cycles to failure																		
	75/ 10,000	…	200/ 60,000	225/ 70,000	250/ 80,000	275/ 90,000	300/ 100,000	325/ 110,000	350/ 120,000	375/ 130,000	400/ 140,000	425/ 150,000	450/ 160,000	475 170,000	500/ 180,000	525/ 190,000	550/ 200,000	575/ 210,000	600/ 220,000
*non-POL-CTRL*	1.0	1.0	0.93 (0.06)	…	0.86 (0.08)	0.73 (0.11)	0.46 (0.12)	0.40 (0.12)	0.33 (0.12)	0.13 (0.08)	0.0	-	-	-	-	-	-	-	-
*non-POL-AB*	1.0	1.0	0.93 (0.06)	…	0.86 (0.08)	0.73 (0.11)	0.66 (0.12)	…	…	0.46 (0.12)	0.40 (0.12)	0.26 (0.11)	0.0	-	-	-	-	-	-
*non-POL-GLZ*	1.0	1.0	1.0	0.93 (0.06)	0.86 (0.08)	…	0.80 (0.10)	0.73 (0.11)	0.46 (0.12)	0.20 (0.10)	0.06 (0.06)	0.0	-	-	-	-	-	-	
*POL-CTRL*	1.0	1.0	1.0	1.0	1.0	0.86 (0.08)	…	…	…	0.73 (0.11)	0.66 (0.12)	0.46 (0.12)	0.26 (0.11)	…	0.20 (0.10)	0.13 (0.08)	0.06 (0.06)	0.0	-
*POL- AB*	1.0	1.0	0.93 (0.06)	…	0.86 (0.08)	…	0.80 (0.10)	0.73 (0.11)	0.66 (0.12)	0.53 (0.12)	…	…	0.40 (0.12)	…	0.33 (0.12)	0.20 (0.10)	0.06 (0.06)	…	0.0
*POL-GLZ*	1.0	1.0	1.0	1.0	1.0	0.86 (0.08)	0.80 (0.10)	…	0.73 (0.11)	0.66 (0.12)	0.60 (0.12)	0.33 (0.12)	0.20 (0.10)	…	0.0	-	-	-	-

Although the internal surface polishing protocol resulted in a controlled material removal of approximately 80 µm, this process primarily aimed to reduce internal defects from the CAD/CAM milling procedure before any surface treatments. The protocol was not intended to be directly applied in clinical practice; further evaluation is necessary. Specifically, crown applications would need to be explored to assess the impact on adaptation and clinical fit. At this stage, the protocol should not be considered a clinical recommendation, and additional studies with sample geometries and clinical conditions are required to confirm its applicability.

This finishing and polishing protocol was specifically developed to assess whether reducing surface defects caused by CAD/CAM burs prior to surface treatments could enhance the mechanical behavior of 4YSZ. Previous studies have indicated that surface treatments alone (such as air abrasion or glazing) are insufficient to completely mitigate these defects^17^. Agreeing with this, our study demonstrated that pre-treatment reduction of milling defects led to improved mechanical performance of 4YSZ. Nevertheless, the clinical feasibility of this approach must be carefully considered, as the procedure could decrease material thickness and compromise marginal and internal fit.

The two proposed surface treatments created different topographies on the 4YSZ surface (Figure 3B, 4C, 4E, and 4F). Air abrasion promotes erosion on the ceramic surface^28^ ^,^ ^29^, where the impact of aluminum oxide particles erodes surface defects^28^. This likely reduces high peaks, resulting in a more uniform distribution of pre-existing defects in both non-POL and POL conditions. In contrast, glaze spray is a surface treatment characterized by its ability to have a healing effect^16^ ^,^ ^17^. Glaze spray acts by partially covering some defects, generating a smoother surface in these areas^16^. However, it tends to accumulate in certain regions, leaving much of the surface uncovered^16^ ^,^ ^17^. Both treatments minimized surface defects, proving more effective in the POL condition groups compared to the non-POL condition, given the application of the finishing and polishing protocol beforehand.

Despite the differences in topography, the surface treatments did not significantly influence the material's flexural strength (Table 3). This is likely because the surface alterations caused by these treatments were less pronounced compared to the changes produced by the finishing and polishing protocol. This is supported by the similar roughness values observed between the POL-CTRL, POL-AB, and POL-GLZ groups (Table 3). Therefore, the finishing and polishing protocol had a greater impact on the mechanical fatigue behavior of 4YSZ than the surface treatments, given the study design and methodology. This is corroborated by a previous study that demonstrated that surface treatments alone, such as air abrasion and glaze, are insufficient to fully address these defects^17^.

In contrast, for the non-POL condition, only the non-POL-AB group demonstrated superior performance compared to the non-POL-CTRL group. This can be attributed to the fact that air abrasion created a surface with more uniform defects and reduced high peaks, which contributed to better stress distribution^29^ (Figure 3A and 4D). On the other hand, in the non-POL-GLZ group, defects that were not fully covered by the glaze likely acted as stress concentrators, leading to early failure^30^. As a result, the mechanical behavior of the non-POL-GLZ group was similar to that of the control group (non-POL-CTRL = non-POL-GLZ; Table 3).

Additionally, it is important to acknowledge some limitations of this study, particularly the challenge of applying glaze to the intaglio surface of a crown in a clinical setting, as glaze accumulation in certain areas could potentially compromise marginal and internal adaptation. This issue requires further investigation to assess its impact on restoration fit. Nonetheless, the trained operator successfully ensured consistent application of the low-fusion porcelain glaze across all groups. The Weibull modulus showed statistically similar values across the groups (Table 3 and Figure 2), indicating consistent structural reliability among all the analyzed groups. This finding suggests that, despite differences in mechanical properties and surface conditions, the fatigue resistance of the tested materials behaved similarly, reflecting comparable structural performance across the different treatments.

Another limitation is that the specimens were simulated rather than milled, which may not fully represent clinical conditions. Despite these limitations, the study provides valuable insights into the fatigue flexural strength of 4YSZ under the tested conditions and is the first to evaluate this combination of factors in a fatigue scenario. Moreover, as only one representative specimen (n=1) per group was analyzed by SEM for topographic visualization, the surface characterization may not adequately reflect the variability within each group. This limitation should be considered when interpreting the qualitative surface findings.

Finally, based on the results, performing an internal finishing and polishing protocol after CAD/CAM grinding significantly improves the flexural strength of 4YSZ. Furthermore, surface treatments such as air abrasion or glaze application result in similar fatigue behavior. However, the finishing and polishing process has a greater influence on the flexural strength of 4YSZ compared to the impact of surface treatments.
